# Mediterranean Diet and Mortality in People with Cardiovascular Disease: A Meta-Analysis of Prospective Cohort Studies

**DOI:** 10.3390/nu13082623

**Published:** 2021-07-29

**Authors:** Chengyao Tang, Xiaowen Wang, Li-Qiang Qin, Jia-Yi Dong

**Affiliations:** 1 Public Health, Department of Social Medicine, Osaka University Graduate School of Medicine, Osaka 565-0871, Japan; chy_tang@outlook.com (C.T.); wangxw@bjmu.edu.cn (X.W.); 2 Department of Epidemiology and Biostatistics, School of Public Health, Peking University Health Science Center, Beijing 100191, China; 3 Department of Nutrition and Food Hygiene, School of Public Health, Soochow University, Suzhou 215000, China; qinliqiang@suda.edu.cn

**Keywords:** mediterranean diet, secondary prevention, cardiovascular disease, mortality, meta-analysis, cohort studies

## Abstract

The association of the Mediterranean diet (MD) with mortality among people with a history of cardiovascular disease (CVD) has not been systematically examined. Hereby, our objective was to investigate the association of MD with all-cause and cardiovascular mortality in people with a history of CVD. We searched five electronic databases including Embase, PubMed, Scopus, Web of Science, and Cochrane Central Register of Controlled Trials to screen eligible studies published before 31 August 2020. A random-effect model was used to examine the association of a 2-unit increment in MD score with the risk of all-cause and cardiovascular mortality. We conducted sensitivity and subgroup analyses and examined potential publication bias by Egger’s and Begg’s tests. Seven cohort studies (eight datasets) with a total of 37,879 participants who had a history of CVD were eligible for the main analysis. The pooled hazard ratios were 0.85 (95% CIs: 0.78–0.93; n = 8) for all-cause mortality and 0.91 (95% CIs; 0.82–1.01; n = 4) for cardiovascular mortality for each 2-unit increment in a score of adherence to MD. Subgroup analyses for all-cause mortality showed that the association appeared relatively stronger in Mediterranean areas (HR = 0.76 [0.69–0.83]) than non-Mediterranean areas (HR = 0.95 [0.93–0.98]) and in studies with a shorter duration (HR = 0.75 [0.66–0.84] for <7 years vs. HR = 0.94 [0.91–0.98] for ≥7 years). No evidence of publication bias was observed. The present meta-analysis of prospective cohort studies provided evidence that adherence to MD improved survival in people with a history of CVD.

## 1. Introduction

Researches on comprehensive dietary patterns provide better insight rather than a single food for understanding the association of diet with risk of chronic diseases [1]. The Mediterranean diet (MD) refers to a traditional dietary pattern of people residing around Mediterranean Sea, and is regarded as one of the typical healthy diet patterns all over the world owing to high consumption of foods abundant in antioxidants and anti-inflammatory nutrients [2]. Widely known characteristics of MD consists of recommendations for high intake of fruits, virgin olive oil, cereals, vegetables especially leafy green vegetables, nuts and legumes, moderate intake of fish, dairy product and red wine, and low intake of sweets [3]. As the currently leading cause of mortality, cardiovascular disease (CVD) results in over 30% deaths around the world [4]. Numbers of observational studies have shown that greater degree of adherence to the Mediterranean diet had an association with a more decreased risk of stroke, coronary heart disease, diabetes, as well as mortality in the general population [5,6,7,8]. For primary prevention, a randomized controlled trial showed that MD or its combination with other diet was associated with a reduction in risk of CVD [9]. In another randomized controlled trial among people at high risk, Estruch et al., reported that MD considerably lowered the risk of main cardiovascular events [10]. Results from clinical trials also supported the favorable effects of MD on cardiovascular risk factors, particularly on blood lipid profile [11].

A second prevention trial showed that MD was effective in preventing cardiovascular death in people with myocardial infarction [12]. During the past decade, evidence from observational studies has grown regarding the health outcomes of MD in secondary prevention. Findings from previous studies were conclusive [13,14,15,16,17,18,19,20,21]. Some, but not all, cohort studies declared that a higher score of MD had an association with a lower risk of mortality after a cardiovascular event. To our knowledge, the advantage of the association between MD and mortality in people with a medical history of any CVD has not been systematically examined. Hereby, our objective was to examine the association of MD with all-cause and cardiovascular mortality among people with a history of any CVD by a meta-analysis. Because the number of clinical trials was limited, we focused on prospective cohort studies.

## 2. Methods

### 2.1. Search Strategy and Data Sources

The present study was conducted and presented as stated in MOOSE guidelines [22]. The study protocol was registered at https://www.crd.york.ac.uk/PROSPERO (Registration No: CRD42020201529). Two authors (C.T. and J.-Y.D.) independently conducted study search, study selection, data extraction, and statistical analysis. The following databases were examined and searched for potentially eligible studies that were published before 31 August 2020: Embase, PubMed, Scopus, Web of Science, and Cochrane Central Register of Controlled Trials. The keywords of exposure, outcome, and study design were used for literature search (detailed in Supplementary Text S1). Moreover, we reviewed the reference list of obtained studies. OpenGray database (www.opengrey.eu) was also searched.

### 2.2. Study Selection

Eligible studies for meta-analysis were those that strictly complied with the following criteria: (1) adherence to traditional or modified MD was analyzed as exposure; (2) the outcome of interest included all-cause or cardiovascular mortality (including coronary heart disease, myocardial infarction, angina, and stroke); (3) a prospective cohort design in which risk estimates with 95% confidence interval (CI) was reported for people with CVD. Any disagreements involved in study selection and inclusion were determined through a consensus meeting.

### 2.3. Data Extraction

We extracted characteristics and results from each study by a standard form. Detailed information consisted of the last name of the first author, publication year, characteristics of the population under study (age, sex, disease history), number of cases and participants, follow-up duration, dietary assessment, death assessment, risk estimates for mortality (95% CI), as well as confounders in the fully adjusted model.

### 2.4. Quality Analysis

The Newcastle–Ottawa scale was utilized to assess the quality of each included study in terms of participant selection, covariate adjustment, and outcome assessment.

### 2.5. Statistical Analysis

In each selected study, the reported relative risks or odds ratios were thought to be equal to hazard ratios (HRs). The risk estimate of mortality for a 2-unit increment of MD score was the main estimate effect of interest in the present study. Values of 0 or 1 were assigned to each indicated component of MD. Individuals who consumed above the sex-specific median value of indicated food components were assigned a value of 1. Summing up these values resulted in a summary score, and a higher score represented a higher degree of adherence to MD. Two studies [18,19] that directly reported an effect size for a 2-unit increment of MD score were directly applied in this meta-analysis. With regard to four studies [14,15,16,17] that reported HRs for categories of adherence to MD, we used generalized least squares [20] for dose–response estimation to convert them for continuous exposure. For one study [13] that reported HR for one-unit increment in the MD score, the reported risk estimates were converted to a 2-unit increment. A random-effect model was used to pool the HRs given the hypothesized presence of heterogeneity [23]. Homogeneity of HRs across studies was assessed using the Q statistic and *I*^2^ [24]. *I*^2^ ≤ 25% indicated low heterogeneity, *I*^2^ = 26–74% indicated moderate heterogeneity, and *I*^2^ ≥ 75% indicated high heterogeneity. We further performed subgroup analysis according to sex, study duration, and study location. Sensitivity analysis was conducted by excluding one study in sequence and then pooling the risk estimates of remaining studies in each turn. Begg’s and Egger’s tests were utilized to examine publication bias. All statistical analyses were conducted in Stata, version 15, and *p* value < 0.05 was regarded as statistically significant.

## 3. Results

**Figure 1:**
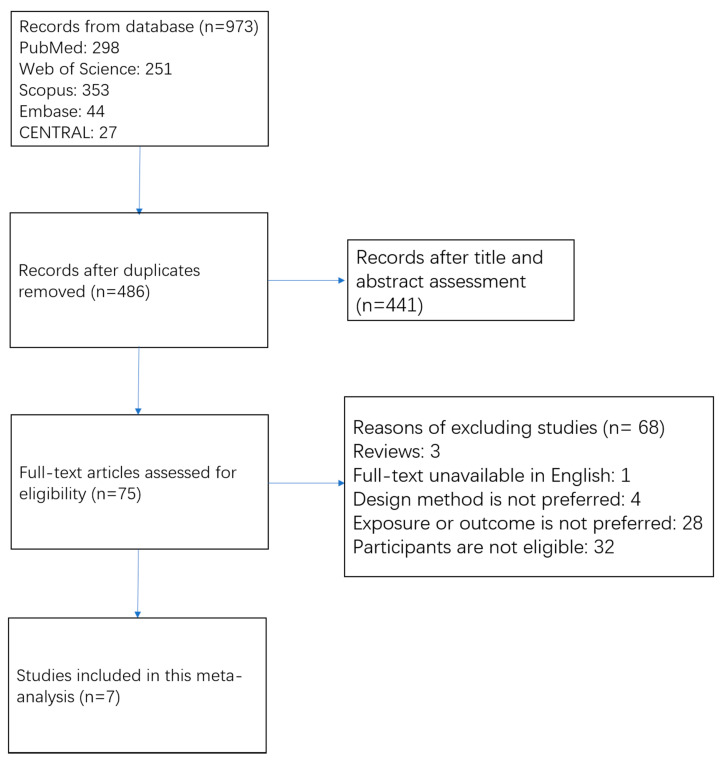
Flow chart of study selection.

The process of study search and study selection is shown in Figure 1. We obtained 973 records from electrical databases. Of them, 487 duplicate articles were first removed. Among the remaining 486 articles, most of them (n = 441) were excluded by scanning titles and abstracts. Frequently excluded reasons included: (1) not cohort study, (2) exposure or outcome not relevant, and (3) no association for people with CVD. A total of 75 studies were assessed for eligibility through a full-text review. Among them, 68 articles were further excluded because full-text articles were unavailable in English (n = 1); study design was review (n = 3); study methods were not prospective cohort study (n = 4); exposures did not include MD, or outcomes did not include all-cause and cardiovascular mortality (n = 28); included participants did not meet the criteria (n = 32). No eligible studies were detected from OpenGrey database. Ultimately, seven prospective cohort studies [13,14,15,16,17,18,19] (8 datasets) were eligible for the meta-analysis.

Characteristics of the selected individual studies are presented in Table 1. The seven selected studies were published from 2003 to 2019. A total of 37,879 participants who had a history of CVD were involved. Five studies [13,14,15,18,19] were conducted in European countries, and the other two [16,17] were carried out in the United States. The follow-up duration lasted from 3.8 to 10.0 years. In terms of study outcomes, all selected studies reported all-cause mortality, and three studies [14,16,19] provided mortality from CVD. All selected studies applied an ordinal scale to assess the adherence to MD. Commonly adjusted covariates included sex, age, body mass index, education status, total energy intake, smoking status, and medical history of hypertension. The results of the quality assessment of each selected study are shown in Online Supplementary Table S1. The scores ranged from 6 to 8 using the NOS scale, indicating a moderate-to-high quality of the original studies.

**Figure 2:**
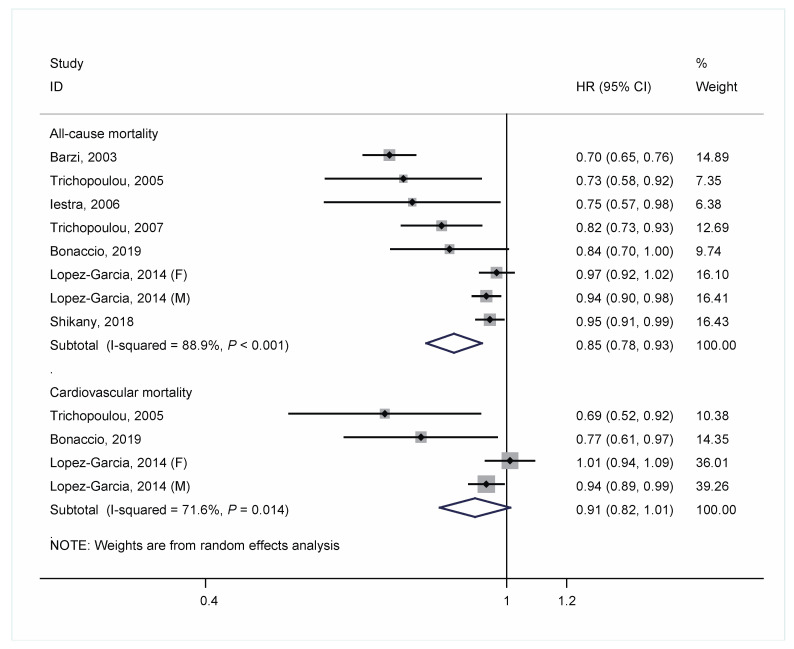
Meta-analysis of associations of each 2-unit increment in a score of adherence to Mediterranean diet with all-cause and cardiovascular mortality in people with a history of cardiovascular disease.

The HRs of each cohort study and the pooled risk estimate are shown in Figure 2. There was a significant 15% decrease in the risk of mortality from all-cause death for each 2-unit increment of MD score (pooled HR = 0.85; 95% CIs: 0.78–0.93), with a high heterogeneity between studies (*I*^2^ = 88.9%; *P*-heterogeneity < 0.001). For risk of mortality from CVD, the pooled HR was 0.91 for each 2-unit increment of MD score (95% CIs; 0.82–1.01; *I*^2^ = 71.6%; *P*-heterogeneity = 0.01). In an additional analysis that combined the HRs for men and women in the study by Lopez-Garcia et al. [16] and then pooled all studies, the overall results changed little.

As shown in the sensitivity analysis, the pooled HR ranged from 0.83 to 0.91 for all-cause mortality and from 0.82 to 0.95 for cardiovascular mortality respectively. No single study was found to have a substantial effect on the overall pooled risk estimates. The results of subgroup analyses of adherence to the MD and risk of all-cause mortality are presented in Table 2. Significant associations were still observed in subgroups of region, study duration, and dietary assessment. The association appeared relatively stronger in Mediterranean areas (HR = 0.76 [0.69–0.83]) than non-Mediterranean areas (HR = 0.95 [0.93–0.98]) and in studies with a shorter duration (HR = 0.75 [0.66–0.84] for <7 years vs. HR = 0.94 [0.91–0.98] for ≥7 years). Of note, significant heterogeneity disappeared among the strata by region. There was no evidence of publication bias for either all-cause or cardiovascular mortality as indicated by the Begg’s test and Egger’s test (All *p* values > 0.10).

## 4. Discussion

In the present meta-analysis of seven prospective cohort studies, a greater degree of adherence to MD had an association with a reduced risk of mortality from all-cause death among populations with CVD. The association appeared evident in Mediterranean areas than that in non-Mediterranean areas. In addition, each 2-unit increment of MD score was associated with a borderline significantly decreased risk of mortality from CVD.

MD is characterized by higher intakes of fruit, vegetables, whole grains, soy, nuts, fish, and olive oil. The potential mechanism for the inverse association of MD with all-cause mortality among patients with CVD might be attributed to these food groups that have beneficial effects on blood pressure, lipid and glucose metabolism, endothelial function, thrombosis, and inflammation, contributing to improved cardiovascular endpoints [25,26]. Furthermore, previous prospective studies have demonstrated that these components are proven to be associated with reduced risks of all-cause and cardiovascular mortality [27,28,29,30].

In the primary prevention of CVD, the significance of MD has been well studied. A previous meta-analysis conducted among general populations free of CVD has shown a 10% (95% CI: 9–11%) reduction in risk of all-cause mortality for a 2-unit increment of MD score [31]. The present meta-analysis extended the findings to the population with a history of CVD and showed a comparable risk reduction. Furthermore, our findings were consistent with the results from randomized controlled trials, which are less susceptible to bias. A clinical trial of 423 patients with myocardial infarction showed that MD had a protective effect on myocardial infarction for persistent 4 years after its first attack [12]. In the same population, MD significantly reduced 37% risk of recurrence and mortality of CVD in 605 patients who had a medical history of coronary heart disease [32]. Moreover, clinical trials demonstrated that adherence to MD improved endothelial function in people with coronary heart disease [33] and reduced the low-density cholesterol atherogenicity in people at high cardiovascular risk [34], thus showing the potential to lower the risk of cardiovascular mortality.

Our subgroup analyses revealed that the association of MD with all-cause mortality among people with a medical history of CVD was relatively stronger in Mediterranean areas (pooled HR = 0.76) than that in non-Mediterranean areas (pooled HR = 0.95). This finding was in accordance with the results from the previous meta-analysis conducted among the general population (pooled HR = 0.82 in Mediterranean populations vs. 0.92 in non-Mediterranean populations) [31]. One possible explanation may be that in the non-Mediterranean area, assessments of adherence to MD were different from the pattern found in Mediterranean areas, in which many people consumed large quantities of legumes, nuts, fruits and vegetables, and olive oil [35]. Additionally, the composition of food groups may be significantly different, owing to diversities in the specific foods within a food group and the different ways of food production and preparation [36]. Meanwhile, the source of alcohol was considered notably different between the two areas when evaluating the adherence to MD [35,36]. Furthermore, the cutoffs of MD score usually use the sex-specific median value for each food component. In different populations, the standard of MD score may differ, thus resulting in misclassification of the degree of adherence to MD score.

In the present study, we observed an inverse, significant association of adherence to MD with mortality from all-cause death, whereas for cardiovascular mortality the association was of borderline significance. The absence of significance for cardiovascular mortality may be due to a limited number of studies and hence weak statistical power for the analysis. Further studies on cardiovascular mortality are therefore needed to verify the role of adherence to MD in secondary prevention of CVD.

As far as we know, the present study is the first meta-analysis to examine the association of adherence to MD with all-cause mortality and cardiovascular mortality in people with CVD. However, several limitations should be noted. First, the quantity of included studies for meta-analysis was relatively limited. Although many prospective cohort studies have examined the association of adherence to MD with risk of mortality, only a few of them concerned populations who were diagnosed with or have a medical history of CVD. Second, despite careful control of covariates in individual cohort studies, residual confounding remained a potential interpretation for the observed results. For instance, the time from the onset of cardiovascular events and treatment for the diseases were not extensively considered in individual cohort studies. Third, relatively high heterogeneity was observed in the main analysis. Differences in population characteristics, food composition, food preparation, and evaluation on the degree of adherence to MD were likely to contribute to the observed heterogeneity. Nevertheless, we were able to detect the source of heterogeneity by the subgroup analysis, which suggested that the study region may be a possible source of inconsistency across studies. Finally, publication bias challenges the validity of any meta-analysis. Nevertheless, no evidence of such bias was observed in our study.

In conclusion, the present meta-analysis of prospective cohort studies provided evidence that adherence to MD may improve survival in people with a history of CVD. Because of the observational nature of included studies, the findings needed to be verified in randomized controlled trials.

## Figures and Tables

**Table 1:** Characteristics of included prospective cohort studies.

Author, Year	Participants	Outcomes	Setting	Duration	MD Assessment
Barzi, 2003	11,323 participants with MI (86% male, mean age 59.4 years)	All-cause death	Italy	6.5 years	An overall MD score ranged from 0 to 10
Bonaccio, 2019	1180 participants with a previous CVD (68% male, mean age 67.7 years)	All-cause and CVD death	Italy	7.9 years	MD score ranged from 0–9
Iestra, 2006	426 participants with a history of MI (67% male, age ≥ 70 years)	All-cause death	Europe	10.0 years	A modified MD Score on an 8-point scale
Lopez-Garcia, 2014	17,415 participants with MI, stroke, angina pectoris, coronary bypass, and coronary angioplasty (35% male, mean age 69 years )	All-cause and CVD death	United States	7.7 years	Alternate MD score ranged from 0–9
Shikany, 2018	3562 participants with existing CHD (61% male, mean age 68.9 years)	All-cause death	United States	7.1 years	MD score ranged from 0–9
Trichopoulou, 2005	1302 Study Participants With CHD at Enrollment (56% male, age range 20–86 years)	All-cause and CVD death	Europe	3.8 years	MD score ranged from 0–9
Trichopoulou, 2007	2671 EPIC-Elderly participants with previous MI at enrolment (69% male, age ≥ 60 years)	All-cause death	Europe	6.7 years	Modified MD score with a 10-point range

CHD, coronary heart disease; CVD, cardiovascular disease; MD, Mediterranean diet; MI, myocardial infarction.

**Table 2:** Subgroup analyses of included studies.

	Datasets (n)	HR (95%CI)	*I*-Square (%)	*P*-Heterogeneity
Total	8	0.85 (0.78, 0.93)	89	<0.01
Sex				
Male	1	0.94 (0.90, 0.98)	-	-
Female	1	0.97 (0.92, 1.02)	-	-
Both	6	0.80 (0.69, 0.93)	90	<0.01
Region				
Mediterranean areas	5	0.76 (0.69, 0.83)	43	0.14
Non-Mediterranean areas	3	0.95 (0.93, 0.98)	0	0.65
Study duration				
<7 years	3	0.75 (0.66, 0.84)	59	0.09
≥7 years	5	0.94 (0.91, 0.98)	29	0.23
Study participants				
<10,000	6	0.89 (0.83, 0.95)	62	0.02
≥10,000	2	0.82 (0.60, 1.14)	98	<0.01
Dietary assessment				
Food frequency questionnaire	4	0.85 (0.76, 0.96)	71	0.02
Others	4	0.84 (0.73, 0.98)	94	<0.01
Mediterranean diet definition				
Traditional score (0–9)	3	0.86 (0.74, 1.00)	67	0.05
Others	5	0.84 (0.74, 0.96)	93	<0.01

CI, confidence interval; HR, hazard ratio.

## Data Availability

Data described in the manuscript, code book, and analytic code will be made available on request.

## Supplementary Materials

The following are available online at https://www.mdpi.com/article/10.3390/nu13082623/s1, Text S1: Methods, Search strategy and Data sources, Table S1: Assessment of study quality using the Newcastle-Ottawa scale.

## Abbreviations

CVD, cardiovascular disease; MD, Mediterranean diet.
