# Supplementary material for: Mediterranean Diet and Mortality in People with Cardiovascular Disease: A Meta-Analysis of Prospective Cohort Studies
Source: Nutrients. 2021 Jul 29;13(8):2623. doi: 10.3390/nu13082623 (PMC8400615; doi:10.3390/nu13082623)
Supplement: Supplementary file 1 [file nutrients-13-02623-s001.zip › nutrients-1287290-supplementary.pdf]

Text S1: Methods

Search strategy and Data sources

The following search syntaxes were utilized in the title/abstract/keywords/medical subject headings: ("Mediterranean Diet" OR "Diets, Mediterranean") AND ("Cardiovascular Disease" OR "Disease, Cardiovascular") AND ("Mortalities" OR "Case Fatality Rate" OR "Age-Specific Death Rate" OR "Death Rate" OR "Mortality Rate") AND ("Cohort Study" OR "Concurrent Study" OR "Follow-up Study" OR "Prospective Study" OR "Longitudinal Study").

Table S1. Assessment of study quality using the Newcastle-Ottawa scale.

Author	Publication Year	Selection	Comparability	Outcome	Total score
Barzi	2003 (6.5y)	☆☆	☆☆	☆☆	6
Bonaccio	2008 (7.9y)	☆☆☆	☆☆	☆☆	7
Iestra	2006 (10y)	☆☆☆	☆☆	☆☆☆	8
Lpoez-Garcia	2014 (7.7y)	☆	☆☆	☆☆☆	6
Shikany	2018 (7.1y)	☆☆☆	☆☆	☆☆☆	8
Trichopoulou	2005 (3.8)	☆☆☆	☆☆	☆☆	7
Trichopoulou	2007 (6.7)	☆☆☆	☆☆	☆☆☆	8

In terms of comparability of each study, all selected studies controlled for sex, age, BMI, and other risk factors related to CVD. As for selection, except one study [13], recruited participants were considered to represent the average population truly or somewhat in the community. All included studies selected the non-exposed cohort drawn from the same community as the exposed cohort study. Four studies[9-11, 14] collected data on diet by using a validated food frequency questionnaire (FFQ), which were considered as high quality on ascertainment of exposure. In one study [12], food consumption data were collected by trained dieticians using a dietary history method, deserving high quality as well. By contrast, the other two studies [13, 16] only utilized simple dietary questionnaire to collect food consumption data of participants. In the present study, we only included those studies, in which included participants were all diagnosed as CVD or had a medical history of CVD. Consequently, outcome of interest was present in all selected studies. Except two studies ([11, 16], the others used clear and validated records or certificates to assess their outcomes. The follow-up of one study [11] was below the adequate follow-up period (5 years). All selected studies reported a relatively small number lost.
